# Treatment of gallbladder stone with common bile duct stones in the laparoscopic era

**DOI:** 10.1186/1471-2482-15-7

**Published:** 2015-01-26

**Authors:** Wei-jie Zhang, Gui-fang Xu, Qin Huang, Kun-lun Luo, Zhi-tao Dong, Jie-ming Li, Guo-zhong Wu, Wen-xian Guan

**Affiliations:** Department of General Surgery, the Affiliated Drum tower Hospital of Nanjing University Medical School, Nanjing, 210008 China; Department of General Surgery, Drum Tower Hospital Clinical College of Nanjing Medical University, Nanjing, 210008 China; Department of Gastroenterology, Affiliated Drum tower Hospital of Nanjing University Medical School, Nanjing, 210008 China; Department of Pathology and Laboratory Medicine, Veterans Affairs Boston Healthcare System and Harvard Medical School, West Roxbury, Boston, Massachusetts 02132 USA; Department of General Surgery, 101st Hospital of PLA, Wuxi, Jiangsu 214044 China

**Keywords:** Cholecystectomy, Laparoscopic, Common bile duct stones (CBDS), Transcystic, Choledochotomy, Primary closure

## Abstract

**Background:**

Laparoscopic common bile duct exploration (LCBDE) for stone can be carried out by either laparoscopic transcystic stone extraction (LTSE) or laparoscopic choledochotomy (LC). It remains unknown as to which approach is optimal for management of gallbladder stone with common bile duct stones (CBDS) in Chinese patients.

**Methods:**

From May 2000 to February 2009, we prospective treated 346 consecutive patients with gallbladder stones and CBDS with laparoscopic cholecystectomy and LCBDE. Intraoperative findings, postoperative complications, postoperative hospital stay and costs were analyzed.

**Results:**

Because of LCBDE failure,16 cases (4.6%) required open surgery. Of 330 successful LCBDE-treated patients, 237 underwent LTSE and 93 required LC. No mortality occurred in either group. The bile duct stone clearance rate was similar in both groups. Patients in the LTSE group were significantly younger and had fewer complications with smaller, fewer stones, shorter operative time and postoperative hospital stays, and lower costs, compared to those in the LC group. Compared with patients with T-tube insertion, patients in the LC group with primary closure had shorter operative time, shorter postoperative hospital stay, and lower costs.

**Conclusions:**

In cases requiring LCBDE, LTSE should be the first choice, whereas LC may be restricted to large, multiple stones. LC with primary closure without external drainage of the CBDS is as effective and safe as the T-tube insertion approach.

## Background

Choledocholithiasis is the second most frequent complication of common bile duct (CBD) diseases and occurs in 10% to 15% of CBD patients [1]. The most common interventional options for patients with choledocholithiasis include: 1) single stage laparoscopic procedure with laparoscopic transcystic stone extraction (LTSE), 2) two-stage approach combining laparoscopic choledochotomy (LC) with pre- or post-operative endoscopic retrograde cholangiography (ERC) [2]. Although both techniques are effective in managing choledocholithiasis, it remains unknown as to selection criteria, complication rate, operative time, post-operative hospital stay, overall cost, morbidity, and mortality. Because of urgent needs, we conducted a prospective comparison study comparing the pros and cons in laparoscopic management of CBD stones between LTSE and LC approaches carried out at a single high-volume tertieary medical center in Nanjing, China.

## Methods

Included in this prospective study cohort were all patients with CBDS and gallbladder stones treated with laparoscopic common bile duct stone extraction (LCBDE) at the Nanjing Drum Tower Hospital and the 101st hospital of the People Liberation Army in China over the period from May 2000 to February 2009. We collected and analyzed patient clinical presentations, laboratory test results, ultrasonography, magnetic resonance cholangiopancreatography (MRCP), or intraoperative cholangiography (IOC). Patient consent for endoscopic surgery and research was obtained before the procedure was started. The study protocol was approved by the Ethics Committee of the Nanjing Drum tower Hospital and the 101st hospital. The protocol was implemented in accordance with provisions of the Declaration of Helsinki and Good Clinical Practice guidelines.

### Surgical procedure

**Figure 1:**
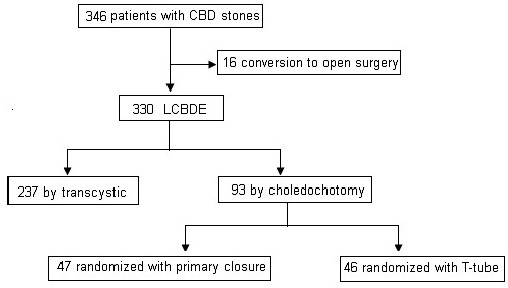
**Trial profile and allocation of patients for LCDBE.**

As described previously, [3] laparoscopic cholecystectomy was performed. The cystic duct was dissected close to the gallbladder and then clipped after identification to prevent stone migration during surgery. Further dissection of the cystic duct was carried out towards the common bile duct in order to IOC and facilitate the introduction offlexible choledochoscopy, which provided the crucial information on stone location, size, number, and the structure of cystic and common bile ducts for the choice between LTSE and LC. The indications for LTSE included stones smaller than 9 mm in size, fewer than 5 in number, and cystic duct lateral entrance to CBD [4–6]. For LC, the indications were: 1) dilated CBD in the diameter of ≥9 mm, stone size ≥ 9 mm, stone number >5, failure of LTSE, and proximal bile ductal calculi [7, 8]. In the LC group, Patients were randomly assigned to either primary closure or T-tube drainage by means of the Research Randomizer ( http://www.randomizer.org/form.htm) (Figure 1).

#### Laparoscopic transcystic stone extraction (LTSE)

In the majority of LTSE patients, the cystic duct was narrow and needed to be dilated. Dilatation was carried out first with blunt, flexible dilators introduced by a 10-mm trocar inserted upright to the cystic duct opening. After dilation, a 5-mm flexible choledochoscope was introduced into the cystic duct. Small stones were flushed out through the papilla.In the majority of cases, stones were extracted with a Dormia basket (Boston Scientific Corporation, USA) under choledochoscopic control. After extraction, a completion cholangiography was performed to detect any upper bile duct stones. If the finding was negative, then the cystic duct was closed with a hem-o-lok clip (Teleflex Medical Inc, USA). Abdominal drainage was not routinely placed unles ssevere acute cholecystitis occurred.

#### Laparoscopic choledochotomy (LC)

The first step of this procedure was to expose the porta hepatis by lifting the round ligament with a transparietal suture and by pulling the cystic duct up and laterally. The anterior aspect of the common bile duct was cleared up over a length of 10 to 20 mm. The LC procedure was performed vertically on the supraduodenal part of the anterior aspect of the common bile duct. All stones visible through the choledochotomy were removed with an atraumatic forceps. Stones located in the lower part of the common bile duct were pushed out through choledochotomy by pressure on the common bile duct wall with blunt forceps or flushed out through the choledochotomy with saline irrigation. The remaining stones were extracted with a Dormia basket under choledochoscopic guidance. Impacted stones were fragmented first by electrohydraulic lithotripsy and then either retrieved with a Dormia basket or pushed out through the papilla. We never dilate the papilla because of the high risk of acute pancreatitis.

After a complete clearance of stones in the CBD, patients were randomized to either primary duct closure or T-tube drainage groups. In the primary closure group, the choledochotomy was closed primarily with 4/0 absorbable sutures (4/0 Vicryl Ethicon, New Jersey, United States) and intracorporeal knotting, whereas in the T-tube drainage group, a latex rubber T-tube in appropriate size (14–20 Fr) was inserted into the CBD incision. After the tube was secured in place, the CBD incision was closed using interrupted sutures (4/0 Vicryl Ethicon, New Jersey, United States). Saline was flushed through the T-tube to rule out leakage.

At the end of the procedure, a single infrahepatic suction drain was placed. This was removed 48–96 h later if there was no bile leakage. Patients in the T-tube group had a cholangiogram on the third to fifth postoperative day. If the finding was normal, the T-tube was clamped and patients were discharged home with the T-tube.

### Discharge and follow-up

The T-tube was removed 3–5 weeks after the operation in the outpatient setting. If there were retained stones, the T-tube was left in place for another 3 to 4 weeks. Patients with primary closure were discharged home once the peritoneal drain was removed. Follow-up assessment using ultrasound was carried out in 3 to 24 months after discharge in the outpatient clinic. If ultra-sound demonstrated possible residual stones, MRCP or Endoscopic Retrograde Cholangiopancreatography (ERCP) was carried out to investigate further.

### Statistical analysis

Patient demographics, stone number, size, location, treatment method, duration of surgery, post-hospital stay, and treatment cost were prospectively collected. Categorical variables were presented as count, and the statistical difference between the two groups was determined by the Chi-square test. Continuous variables were expressed as mean ± standard derivation (SD) and compared with the Student’s *t* or the Mann–Whitney test. Statistical significance was determined by the *p* value less than 0.05. All analyses were carried out with Statistical Package for the Social Sciences (SPSS 12.0 for Windows, Chicago, United States).

## Results

### Reasons for conversion

Included in the cohort were 346 consecutive patients with common bile duct stones, 16 (4.6%) of whom were converted to open surgery because of the following conditions: 1) the narrow or tortuous cystic duct, 2) dense fibrotic adhesion with obscured anatomy, 3) impacted stone, 4) laparoscopic failure, 5) rupture of the cystic duct, 6) intrahepatic stone, 7) cholecystoduodenal fistula, 8) gallbladder bed bleeding, and 9) duodenum injury (Table 1). In The remaining 330 patients, 237 underwent LTSE and 93 required LC. In the LC group, all patients were randomly assigned to either the primary closure (n = 47) or the T-tube drainage (n = 46) groups.

**Table 1:** **Reasons for conversion to open common bile duct exploration in 16 patients**

Reason for conversion	Patients (n)
Adhesions and unclear anatomy^#^	5
Technical failure*	4
Impacted stone	3
Rupture of cystic duct	1
Cholecystoduodenal fistula	1
Gallbladder bed bleeding	1
Duodenum injury	1

^#^Including three patients with too narrow or tortuous cystic duct.

*including one patient with intrahepatic stone.

### Stone clearance

A stone retrieval basket was used for bile duct stone clearance. One-stage stone clearance was successful in 312 of 330 patients (94.5%) but failed in 17 patients who were treated successfully by postoperative ERCP and stone extraction. Only one case in the T-tube drainage group underwent postoperative cholangiography. The retained stones were removed through the sinus tract of the T-tube using a choledochoscope.

### Morbidity

There were two bile duct injuries sustained in the whole series, but no mortality occurred. There were, not surprisingly, more complications in the LC group, compared to the LTSE group (Table 2). The most common complications were biliary complications (15/237 vs 14/93), which including retained stone (13 cases), bile leakage (10 cases), acute pancreatitis (2 cases), bile duct injury (2 cases) and acute biliary peritonitis after T-tube removal (2 cases), and secondly wound infections (5/237 vs 3/93). The overall complication rate was 16.7% (31/237 vs 24/93) (Table 3).

**Table 2:** **Complication rates for patients after LCBDE via transcystic approach versus choledochotomy**

Complications	Transcystic	Choledochotomy	***P*** Value
	(n = 237)	(n = 93)	
**Biliary complications**	15	14	**0.012**
Acute pancreatitis	1	1	
Bile duct injury	2	0	
Bile leakage	3	7	
Acute biliary peritonitis after T-tube removal	NA	2	
Retained stone	9	4	
**Other complications**	16	10	0.225
Umbilical hematoma	4	1	
Ileus	4	0	
Respiratory complication	3	3	
Wound infection	5	3	
Peritubal infection	NA	3	
**Total complications**	31	24	**0.005**

*Values that were significant are in boldface. NA: not available.

**Table 3:** **Comparison of postoperative complications between Primary closure versus T-tube drainage underwent choledochotomy**

	Primary closure	T-tube	***P*** Value
	(n = 47)	(n = 46)	
**Biliary complications**	6	8	0.533
Bile leakage			0.688
Major biliary leakage (≥100 ml/24 h)	1	3	0.594
Minor biliary leakage (<100 ml/24 h)	1	2	1
Complications related to T-tube	NA	2	-
Acute pancreatitis	1	0	1
Retained stone	3	1	0.625
**Other complications**	3	7	0.180
Total complications	9	15	0.138

### Transcystic approach versus choledochotomy

Laparoscopic common bile duct exploration was performed through either the transcystic (LTSE) approach in 237 patients (71.8%) or choledochotomy (LC) in 93 patients (28.2%). Comparison in patient demographics and clinical outcomes between LTSE and LC groups was presented in Table 4. Compared to the LC group, patients in the LTSE group were significantly younger, had smaller and fewer stones, and experienced fewer complications. Consequently, the operating time and postoperative hospital stay were significantly shorter in the LTSE than in the LC group. Therefore, the overall cost was significantly lower in the LTSE than in the LC group. However, the stone clearance rate and the frequency of conversion to open surgery were similar between the two groups. In the LTSE group, the stone removal success rate was 96.2% (228/237) and only 9 (3.8%) failed and were converted to endoscopic sphincterotomy or endosiopic papillary balloon dilation. Similarly, the stone clearance rate was 95.7% in the LC group and only 3 (4.3%) required endoscopic spincterotomy to remove stones through the sinus tract of the T-tube using a choledochoscope.

**Table 4:** **Patient demographics and clinical outcome data after LCBDE via transcystic versus choledochotomy approach**

	Transcystic	Choledochotomy	***P*** Value
	(n = 237)	(n = 93)	
Sex			0.062
Male	98	49	
Female	139	44	
Age (years)	54.7 ± 13.3	54.2 ± 16.3	**0.003**
Acute cholecystitis	23	9	0.757
Number of CBDS	3.2 ± 1.8	4.5 ± 2.6	**<0.0001**
Diameter of CBDS (mm)	5.3 ± 2.1	12.0 ± 3.5	**<0.0001**
Operating time (min)	76.0 ± 20.2	116.1 ± 28.1	**0.040**
Stone clearance	228	89	0.832
Conversion to open surgery	11	5	0.780
Postoperative complications	32	24	**0.012**
Hospital expenses (RMB)	7435.3 ± 994.8	10968.7 ± 1156.4	**0.008**
Postoperative hospital stay (days)	3.9 ± 1.8	6.7 ± 2.8	**0.019**

LCBDE, Laparoscopic common bile duct exploration CBDS, common bile duct stones.

Values that were significant are in boldface.

### Outcome comparison between primary closure and T-tube drainage

For the LC patients, demographic characteristics and clinical presentations of common bile duct stones were similar between the primary closure group and the T-tube drainage group. There was no statistically significant difference in stone size, number, clearance rate, and postoperative complications between the two groups. However, the endoscopic procedure time, post-operative hospital stay and cost were significantly lower in the primary closure group than in the T-tube drainage group (Table 5). In the primary closure group, the surgical success rate was 93.6% (44/47), and only 3 cases required endoscopic sphinctotomy. In contrast, the surgical success rate was 97.8% in the T-tube drainage group with only one case converted to postoperative cholangiograhy. In cases with large and impacted ampullary stones, patients were treated with percutaneous endoscopic holmium laser lithotripsy as *Healy et al.* describled [9].

**Table 5:** **Comparison of surgical results between primary closure and T-tube drainage group**

	Primary closure	T-tube	***P*** Value
	(n = 47)	(n = 46)	
Sex			0.593
Male	22	19	
Female	25	27	
Age (years)	52.3 ± 16.6	52.0 ± 15.9	0.533
Acute cholecystitis	4	5	0.973
Diameter of CBDS (mm)	11.9 ± 2.7	12.3 ± 3.4	0.058
Number of CBDS	4.8 ± 2.7	4.2 ± 2.4	0.468
Operating time (min)	106.0 ± 22.6	126.4 ± 29.5	**0.022**
Stone clearance	44	45	0.846
Conversion to open surgery	2	3	0.980
Postoperative complications	9	15	0.138
Hospital expenses (RMB)	10317.3 ± 735.8	11634.3 ± 1133.3	**0.034**
Postoperative hospital stay (days)	5.1 ± 1.6	8.4 ± 2.8	**0.025**

Values that were significant are in boldface.

During the follow-up period, only two patients lost (0.84%). Recurrent CBD stones were found in only 6 (6/318, 1.9%) patients in which 3 were in the LTSE group, 1 in the T-tube group, and 2 in the primary closure.

## Discussion

Since the advent of laparoscopic cholecystectomy, the management strategy for CBD stones has been a subject of much discussion but with the absence of an established consensus.

There were several methods in the management of patients with choledocholithiasis: Single stage laparoscopic procedures, two stage methods combining LC with pre- or post-operative ERC. For the single stage laparoscopic procedures, LC can be combined with laparoscopic exploration of the common bile duct, either as a choledochotomy or as a LTSE procedure. Preoperative Endoscopic sphincterotomy (EST) has been the procedure of choice for most physicians [10, 11]. Although the success rate for stone clearance equals 87% to 97%, ERCP and EST are associated with morbidity and mortality rates of 5% to 11% and 0.77% to 1.2%, respectively [12–15].

According to some randomized studies, the single-stage technique has been shown to have the advantages of shorter hospital stay and lower postoperative morbidity [16–18]. The present study showed that LTSE was safe withthe postoperative complication rate of 13.5%. Bile duct stone clearance was was successful in 96.2% of LTSE patients, similar to that of LC cases. Compared to the LC group, the operating time and postoperative hospital stay were shorter and the expense was lower in the LTSE group. Total complication and biliary complication rates in were also significantly lower in the LTSE group than in the LC group.

Although attractive, the LTSE approach was confined to CBD stones smaller than 9 mm in size, fewer than 5 stones, and stone location in the CBD distal to the cystic duct confluence. If these criteria were not fulfilled, or the LTSE approach failed, LC had to be used [4–8].

Laparoscopic primary closure of CBD is safe and effective for the management of CBD stones, and can be performed routinely as an alternative to T-tube drainage [7, 19]. In our study, LC cases were randomized to either the T-tube or the primary closure groups. The the operation time and postoperative hospital stay were shorter and the hospital expenses lower in the primary closure group than in the T-tube group. We have shown fewer, but no statistical, complications in the group with the primary closure.

For Overall, the postoperative complication rate, in the primary closure group was insignificantly lower than that in the T-tube group. Similar to the findings reported previously [20], the most complication in the T-tube group in our study was related to the use of the T-tube. Therefore, postoperative T-tube drainage is unnecessary for decompression of the biliary tree. In addition, the use of intraoperative choledochoscopy and cholangiography can also help eliminate the overlooked biliary tree diseases.

The length of hospital stay was shorter in the primary group than in the T-tube group. We believe that the risks of dehydration and saline depletion in patients with open T-tubes at home are contraindications to discharge. Therefore, the patients need to keep their T-tube in the hospital until after the clearance by the T-tube cholangiogram. It is unacceptable to the majority of our patients to go home with a functioning T-tube. Prolonging hospital stay would not only increase the total hospital expense, but also raise the risk of complications and the need for transfusion.

## Conclusions

LTSE and LC with bile duct stone extraction can be performed with high efficiency, minimal morbidity and without mortality. LTSE is feasible and should be chosen as the first therapeutics, whereas LC should be restricted to large, multiple stones that cannot be extracted through the cystic duct. Postoperative T-tube drainage is unnecessary for decompression of the biliary tree.

## Abbreviations

CBD: Common bile duct
LTSE: Laparoscopic transcystic stone extraction
LC: Laparoscopic choledochotomy
ERC: Endoscopic retrograde cholangiography
LCBDE: Laparoscopic common bile duct stone extraction
MRCP: Magnetic resonance cholangiopancreatography
IOC: Intraoperative cholangiography
ERCP: Endoscopic retrograde cholangiopancreatography
EST: Endoscopic sphincterotomy.
